# Tools for Ground-Truth-Free Passive Client Density Mapping in MAC-Randomized Outdoor WiFi Networks

**DOI:** 10.3390/s23136142

**Published:** 2023-07-04

**Authors:** Feifei Yang, Iness Ahriz, Bruce Denby

**Affiliations:** 1 Aleia, 75008 Paris, France; feifei.yang@espci.fr; 2 Institut Langevin, ESPCI Paris, PSL University, CNRS, Sorbonne Université, 75005 Paris, France; 3 C.N.A.M. (Conservatoire National des Arts et Métiers), 75003 Paris, France; iness.ahriz@cnam.fr

**Keywords:** audience monitoring, GDPR, MAC randomization, probe request, WiFi, localization

## Abstract

In the past few years, data privacy legislation has hampered the ability of WiFi network operators to count and map client activity for commercial and security purposes. Indeed, since client device MAC devices are now randomized at each transmission, aggregating client activity using management frames such as Probe Requests, as has been common practice in the past, becomes problematic. Recently, researchers have demonstrated that, statistically, client counts are roughly proportional to raw Probe Request counts, thus somewhat alleviating the client counting problem, even if, in most cases, ground truth measurements from alternate sensors such as cameras are necessary to establish this proportionality. Nevertheless, localizing randomized MAC clients at a network site is currently an unsolved problem. In this work, we propose a set of nine tools for extending the proportionality between client counts and Probe Requests to the mapping of client densities in real-world outdoor WiFi networks without the need for ground truth measurements. The purpose of the proposed toolkit is to transform raw, randomized MAC Probe Request counts into a density map calibrated to an estimated number of clients at each position.

## 1. Introduction

Since the introduction of commercial WiFi networks over twenty years ago, there has been considerable interest in determining the locations of users of the service for commercial purposes such as client counting and monitoring, as well as for safety and security concerns. Localization is typically performed by leveraging the physical properties of a client’s WiFi signals, such as the Received Signal Strength (RSS), at several network Access Points (APs). Most systems make use of the abundant Probe Request (PR) frames emitted regularly by client devices even when not connected to a network [1,2,3,4,5]. Sophisticated WiFi client localization interfaces have now become commonplace worldwide. Section 1.1, Section 1.2, Section 1.3, Section 1.4 and Section 1.5 provide historical and technological background on the current status of the field.

### 1.1. Client Monitoring for Business Metrics

Footfall, also known as client counting or simply traffic, is a measure of the number of visitors entering a commercial site, such as a retail shop, commercial center, museum, and so on. Footfall counts have been used for decades to help retailers and site managers gauge the attractiveness of their offers and improve the overall customer experience. Initially an affair of simple hand clickers, turnstiles, or weight-sensitive mats placed in doorways, footfall has evolved into what is today considered a key business metric that is usually obtained using more sophisticated techniques such as still and video surveillance cameras, Passive InfraRed (PIR) thermal detectors, active infrared Time of Flight (ToF) imagers, WiFi, and others. Data from the sensors is typically transferred via an internet protocol to be stored and analyzed on a server. Realtime footfall statistics have now become a valuable business analytics input, useful not only for retail and marketing analysis, but also in so-called Smart Building and Smart City applications, including energy use monitoring, people flows, and safety and security concerns.

### 1.2. Wireless Client Monitoring

While radio-based client-held devices such as audio-guides have been in use for a number of years, when cellphones became popular in the 1990s, interest in exploiting the connectivity of these devices for footfall applications began to grow. With the arrival of smartphones and their nearly unanimous adoption by consumers, however, the prospect of an active WiFi/Bluetooth Network Interface Card (NIC) in the pocket of every single customer led to a paradigm shift in mobile client monitoring. Indeed, while many of the footfall tools mentioned in the previous section are still alive and well, there has been a veritable explosion in the use of wireless technologies for collecting client activity data. Beyond simple client counting, wireless also allows a means of localizing clients via an analysis of the strength of their radio signals. Thus, by leveraging ubiquitous internet-enabled client devices and an existing wireless network, wireless client monitoring can be much less dependent on specialized hardware, novel expertise, and labor-intensive deployment, calibration, operation, and maintenance, when compared to surveillance cameras, infrared sensors, or legacy footfall solutions.

Driven by a desire for ubiquitous connectivity, Wireless Local Area Networks (WLAN) using WiFi, or in some cases, Bluetooth technologies, are today found in almost all public commercial areas, with city-wide WiFi coverage rapidly becoming the norm. Many of the well-known personal localization services, such as Google Maps and others, exploit existing WiFi and Bluetooth networks, together with GPS signals, for navigation and orientation in indoor and outdoor spaces. From the standpoint of client monitoring, however, it is generally the WiFi signals generated by the smartphones themselves, captured at the network APs, that are of principal interest for counting and localization purposes.

Indeed, in order to assure rapid access to the ambient wireless networks, the WiFi NICs found in smartphones emit on a regular basis certain Control Frames that establish a list of available networks and announce the device’s presence and its capabilities. Of particular interest is the Probe Request (PR) frame, mentioned earlier, emitted on the order of 500 times in a 24 h period by a typical NIC [1,2], even when not connected to a network, that identifies the client to the service provider via the NIC’s unique Medium Access Address, or MAC. Nowadays, all major wireless network manufacturers, such as Cisco/Meraki and CommScope/Ruckus, to name just two, commercialize sophisticated, turnkey WiFi client monitoring and market analysis solutions for enterprises, commercial centers, Smart Cities, etc., based on this model. Such systems feature real-time graphical interfaces that display estimated client counts, traffic statistics, management parameters, etc., for the different zones covered by the underlying network, providing footfall as well as other business analytics of interest to the business managers deploying the software.

### 1.3. Data Privacy

Since 2016, however, with the advent of the European General Data Privacy Regulation, or GDPR, and similar legislation elsewhere in the world, many of the opportunities for WiFi client monitoring and localization have been jeopardized. To preserve clients’ identities, Medium Access Control addresses are nowadays considered private information and must be anonymized using a non-reversible hash function. As a given MAC address nevertheless always hashes to the same token value, for further protection, all user WiFi devices being manufactured today also attribute a new, randomized MAC address with each transmission. WiFi service providers, consequently, are now faced with a plethora of PR signals without any possibility of correlating them with the different clients, thus rendering invalid the de-facto industry-standard wireless client monitoring model just described.

### 1.4. Counting Randomized MAC WiFi Clients

Workarounds to the problem do exist. One is to use clues in the digital content of received frames to effectively de-randomize PRs. A second is to concentrate on connected clients who, by subscribing to the WiFi service, have consented to the use of their private fixed MAC address. Both have important drawbacks, as will be discussed in Section 2.

Beyond the workarounds, though, in the past few years, researchers have developed methods of estimating genuine client counts from MAC-randomized PR using statistical methods. It is found that for sufficiently large client populations, the number of PRs is linearly proportional to the true number of clients. The problem remaining then amounts to determining the appropriate proportionality constant, which can be achieved either by comparison to a ground truth obtained from other sensors, such as cameras, entry monitors, etc., as in [6,7,8], or without ground truth, albeit with reduced accuracy, by exploiting observed periodicities in plots of PR counts versus time [1]. This type of approach forms the starting point for the mobile client localization techniques that will be developed in the present work.

### 1.5. Localizing Anonymized WiFi Clients

If the problem of *counting* MAC-randomized clients is perhaps in the process of being solved, following the observations in Section 1.4, assigning a *location* to a group of anonymized clients presents a number of additional difficulties. Localization in wireless networks has been an active field for a number of years, with a wide variety of techniques proposed [9,10,11,12,13]. Using unassisted WiFi, positioning accuracies of about 2 m in indoor scenarios or 10 m outdoors are possible when an adequate number of APs are involved. A simple, popular, and effective method of localization in outdoor WiFi networks that adapts readily to WiFi localization of smartphones is triangulation, in which a user’s position is calculated from RSS values received simultaneously at three or more APs. Triangulation is actually possible for a single randomized MAC PR since all APs receiving the PR produce identical hash tokens. In practice, though, in many outdoor networks today, the main focus is on providing connectivity, while the ability to also perform localization—which is more expensive since it requires higher AP redundancy—remains of secondary importance. In such a case, a substantial fraction of emitted PRs may be captured by only one or two APs, making triangulation impossible. Furthermore, subsequent emissions will carry new, unseen MAC address tokens, making it impossible to know if they arise from the same device as the previous PR or from a different client.

Indeed, while positioning is by nature sub-optimal in networks where localization was not an initial design priority, under GDPR, standard solutions become a priori impossible for any but connected subscribers having fixed MAC addresses. As a result, tools for performing localization despite these challenges, are today in substantial demand. The goal of the present article is to propose a set of tools intended as a first step towards re-establishing client monitoring for commercial and security purposes in an era when many traditional approaches are no longer available.

### 1.6. A Proposed Toolbox

The article presents a set of nine tools designed to facilitate the preprocessing, display, interpretation, localization, and counting of outdoor WiFi network clients based on raw randomized MAC PR counts. The objective of the toolkit is to transform randomized MAC Probe Request counts into a density map calibrated to the number of clients at each position. In many instances, the mathematical machinery deployed in the tools is well established. The contribution of the proposed work lies in assembling the necessary elements into a coherent package to make sense of a complicated problem that many network operators have dismissed as intractable. The toolkit contains the following:

- Preprocessing Tool for selecting client populations for study;
- “Bowl” Tool for displaying localization probability densities;
- Renormalization Tool for condensing distributed probabilities into decision regions;
- Counting Tool for estimating the proportionality between PRs and clients;
- Filtering Tool for removing peaks in the density that obscure smaller scale structures;
- Localization Tool for device localization and calibration of the propagation model;
- Fiducial Tool for delineating client density regions;
- Drill-Down Tool for the study of client densities on different scales;
- Re-assembly Tool to create the final map calibrated for clients rather than PR.

Some of these are straightforward procedures, where the word “tool” may seem excessive; for others, more involved, the label indeed applies. The “Bowl,” Filtering, and Drilldown tools, for instance, employ relatively standard graphical methods, whereas the Fiducial, Localization, Counting, and Renormalization tools are innovative and embody much of the originality of the present work.

The remainder of the article is structured as follows. The next section gives an overview of state-of-the-art WiFi networks in the era of GDPR and introduces the use of PR for client monitoring. The problem of IoT in WiFi networks and the definition of the client classes used in the article are provided in Section 3. Section 4 is a tutorial introduction to the tools proposed, highlighting the ways in which they address problems of real-world WiFi networks as well as the challenges posed by GDPR rules. Section 5 and Section 6 showcase the proposed localization tools through examples drawn from two real-world outdoor WiFi datasets. Discussion, conclusions, and possible future directions appear in the final section. Because of the tutorial nature of the article, certain techniques are first introduced in a didactic fashion and returned to in detail in subsequent sections.

## 2. Related Work

As presented in Section 1.2, commercial WiFi localization solutions furnishing position-dependent client analytics are today quite sophisticated; however, since the advent of the GDPR, they are applicable only to clients who have formally subscribed to the WiFi service and thus authorized the use of their private fixed MAC address.

As mentioned in Section 1.4, a way around this problem that has garnered considerable attention is the so-called de-randomization approach for PRs. In this approach [14,15,16,17,18,19,20,21,22,23,24,25,26], information from digital frame content such as the frame Sequence Number, particular Information Elements (IE), Preferred Network List, and certain time delay measurements derived from these quantities can be analyzed statistically to group together PRs arising from a unique client, thus undoing the effect of the MAC randomization. Though rigorous and often quite effective, a recognized drawback of this approach is that wireless device manufacturers are perpetually on the lookout for such security loopholes, which the next generation of devices is likely to close. A consensus is growing that data privacy is here to stay and that researchers would do better to concentrate on intrinsically non-device-specific privacy-preserving approaches. Indeed, this is the philosophy adopted in the present work as well as in other recent contributions [1,6,7,8].

A second workaround amounts to monitoring only connected clients or others with fixed MAC addresses. This choice, however, comes at a heavy price. In outdoor WiFi networks, only a small percentage of clients nowadays choose to subscribe to the service, preferring to rely on cellular telephone connectivity to access the internet. Indeed, the authors of [5] report a fivefold increase in the percentage of randomized MAC clients, from 10% to 50%, between the years 2016 and 2018. In [1], fixed-MAC clients in 2020–2021 are found not only to represent less than 10% of all PRs, but also to have significantly different PR statistics compared to randomized MAC clients. This latter effect is explained by the fact that a growing fraction of fixed MAC devices correspond either to Internet of Things (IoT) devices or to human clients engaged in extended services such as Peer-to-Peer, both of which produce uncharacteristically large numbers of PR [1].

A variant of this second workaround is to assume that the number of randomized MAC clients amounts to some fixed fraction of the total number of clients. The authors of [27], for example, are able to present a global chi-squared approach to outdoor localization, which in fact shares some elements of the tools presented here but treats randomized MAC clients as an add-on to their analysis of fixed-address clients. For the reasons already stated, however, this approach becomes less relevant as the percentage of randomized MAC clients grows.

Again, as introduced in Section 1.4, it has recently been demonstrated theoretically in [1,6] and observed empirically in [1,6,8] that due to the statistical independence of the client density and client device emission probability distributions, a simple proportionality exists, in the limit of a large number of clients, between the number of randomized MAC clients, C, and the corresponding number of randomized MAC PR, P, that is, C = P/X, where the proportionality constant X is understood to be the mean device PR emission probability for the time window concerned. This observation is currently the key to understanding client numbers and client densities in the era of data privacy. It will be liberally exploited in this work.

Though site-dependent, X for a time window of one day turns out to be of the order of 500, as alluded to in Section 1.2. On the one hand, the appropriate value of X for a particular site can be obtained using ground truth from alternate counting systems, such as cameras. This was the technique employed in [6,8] for indoor localization in networks having relatively high AP density and equipped with complementary ground truth sensors located in the various zones covered by the WiFi network.

A second technique for obtaining X, albeit with reduced precision, is by estimating it statistically from periodicities observed in the distribution of PR counts versus time, as was reported in [1] for several low-AP count outdoor scenarios. The independence of ground truth of this second method, despite its lesser precision, is quite interesting and will be exploited in the present work. Indeed, the principal innovation in the present work is to extend ground truth-free methods from simple client counting to outdoor client density mapping, calibrated not in PR but in clients, based entirely on readily measurable quantities and not requiring any auxiliary sensors, by employing a novel set of tools and the instructions for applying them.

## 3. Datasets and Definitions

The proposed toolbox is presented first using the data sets used and some necessary definitions.

### 3.1. Outdoor WiFi Network Data Sets Studied

The datasets studied include detailed, time-stamped PR records from public outdoor WiFi networks at an outdoor tourist site, Site 1, and a campground, Site 2, both in France. They are part of a larger data set, including the French city data introduced in [1]. Note that for client counting purposes, there are some important differences between the present data and the city data of [1] (to be discussed in Section 3.2 and Section 6.1). As such, the two studies are complementary. The data presented here were accumulated from the last week of March to the end of June in 2021, giving total PR counts of 7.0 million for Site 1 and 5.3 million for Site 2. A separate file stores connection detail records for clients who have at some point formally signed into the WiFi service. The AP layouts of the sites appear in the figures in the following sections. In accordance with the current European data privacy legislation, all client MAC addresses are replaced by anonymized hash-coded strings.

### 3.2. Client Classes

By counting the number of times in a selected time window that a distinct anonymized MAC is seen and comparing it to a threshold, and by also taking into account the connection details file, PRs are identified as belonging to one of three classes:

- Client Randomized, CR
  The MAC string is seen from 1 to “threshold” times.
- Client Fixed, CF
  The MAC string is seen more than “threshold” times.
- Client Connected, CC
  The MAC string appears in the connection details file.

Connected clients are separated out first, so that the classes are mutually exclusive. These client class definitions are the same as those introduced in [1]. An important difference is that a threshold of 3 is chosen here, rather than 2 as in [1], which will turn out to be a key element in some of the tools presented.

### 3.3. A Caveat: OUI and IoT

Despite randomization of the MAC, its first 3 bytes, called the Organizationally Unique Identifier, or OUI, are retained and become part of a PR entry in the data record. The OUI is intended to provide information on the manufacturer of the WiFi network card producing the PR, available by interrogating a public OUI web repository. In practice, OUI responses include a baffling array of manufacturers that are sometimes difficult to clearly identify. A detailed investigation [1] suggests up to 80% are manufacturers of IoT devices such as lighting, heating, cameras, etc., whose network behavior is quite distinct from that of true client devices. These observations, aside from the issue of GDPR, provide another strong motivation for basing crowd estimation solutions exclusively on CR-class clients, as these almost certainly correspond to bona fide clients. In this article, all density estimates are based on CR.

### 3.4. Preprocessing Tool

To prepare the data for the remaining steps in the toolbox, it is necessary to carry out a preprocessing step based on the points outlined in Section 3.1, Section 3.2 and Section 3.3. In this article, a baseline time window of three hours was chosen, long enough to provide a statistically rich sample of thousands of PRs, yet short enough to enable following client behavior over the course of a day. The CR category of client PRs is first selected by applying a threshold of 3 to the number of times the PR MAC address appears in the selected data sample. This eliminates subscribed clients as well as IoT devices, producing a sample of P randomized MAC PRs that have each been seen one, two, or three times. As mentioned, P is usually a number in the thousands here, which means that the huge bulk of MAC addresses are all different due to the randomization at each new emission. Next counted is the number of PRs seen three times in this sample, P_3_, after verifying that their timestamps are consistent with near-simultaneous reception. We recall that the MAC of a PR received at multiple APs will hash to the same result at all APs concerned. The quantities P and P_3_ will be important later in the localization, counting, and reassembly steps.

## 4. Tutorial Introduction to the Proposed Toolbox

This section presents a tutorial-style introduction to some of the difficulties encountered in real-world outdoor WiFi network localization and the manner in which the proposed tools can be brought to bear upon them. Note that the Preprocessing Tool was already introduced in Section 3, while the treatment of the Fiducial, Drill-Down, and Reassembly Tools will be reserved for Section 5 and Section 6. The section begins with an introduction to triangulation using received client RSS values.

### 4.1. Basics of Triangulation, Expected Positioning Accuracy

As outdoor WiFi networks cover extended areas, obtaining full coverage for both traffic and localization is challenging. Numerous approaches based on RSS [9], Time of Arrival (ToA) and its variants, or Channel State Information (CSI) are possible, either in fingerprint or in ranging modes [10,12], or combined with GPS [13]. However, because of its simplicity and widespread use, the focus here is on triangulation, also referred to as trilateration.

The physical origin of the triangulation approach is embodied in the well-known Friis equation relating RSS with distance:

(1) $$\mathrm{RSS}\left(\mathrm{dBm}\right)=A\left(\mathrm{dBm}\right)+10n\log_{10}\left(d\left(m\right)\right)$$

Here, RSS is measured in dB with respect to 1 milliwatt (dBm), while the client device-AP distance d is in meters. The constant A, also in dBm, is the emitted power at 1 m from the client device—by industry standard, normally taken as −30 dBm. The constant *n* is called the fading exponent. The Friis equation can be derived from first principles for free space propagation, where power decreases as the inverse square of the distance, giving *n* = 2. Propagation over a flat, unencumbered surface can also be shown to produce *n* = 4. For intermediate situations with clutter and multipath effects, as expected in outdoor WiFi networks, values in the range 2–3 are encountered, depending on the particular environment.

Concerning positioning accuracy, while in indoor WiFi networks, where AP counts are higher, a precision of ± a few meters is typical, in a calibrated outdoor network, an accuracy of ±10 m is already considered a good result. This observation will be an important consideration in evaluating the viability of some of the tools proposed in the article.

### 4.2. Bowl Tool

**Figure 1:**
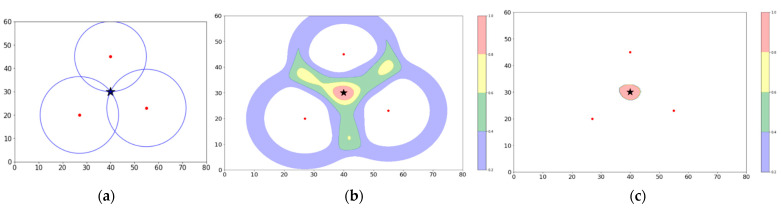
(**a**) Triangulation of a single PR using circles determined from RSS. Red dots are AP positions. The solution is indicated by the black star. (**b**) Equivalent method using the sum of probability “bowls” (see text) to localize the PR. The black star coincides with the high-probability zone in red. (**c**) Reincorporation of all probabilities into the red “decision zone”.

Let us first consider a toy configuration with three APs in a triangular arrangement as in Figure 1a. A client device somewhere within the triangle emits a PR that propagates outward and is captured by each of the three APs in turn to produce RSS1, RSS2, and RSS3. By inverting the Friis equation, one can calculate the distances R1, R2, and R3 from the client device to each of the APs. By tracing circles with these radii, as in the figure, the client device is located at the point where they intersect, indicated by a black star.

**Figure 2:**
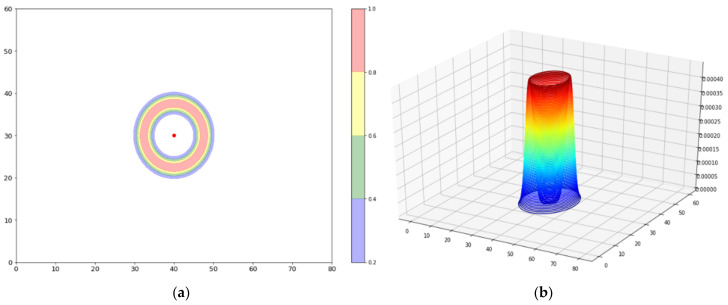
Illustration of the bowl formula (**a**) in 2D projection and (**b**) in 3D.

An equivalent but somewhat more realistic simulation is shown in Figure 1b. In this case, the anticipated uncertainty in measured RSS values is taken into account, which is conventionally taken to have a Gaussian distribution with a variance of 2 dB. In this case, given an RSS, a PR produces a spatial probability density distribution in the form of a “bowl” whose radius is calculated using Friis and whose “lip thickness” is dictated by the 2 dB RSS uncertainty, translated, again through Friis, into a physical distance. The formula for the bowl is then given by

(2) $${\hat{f}}_{p}\left(x,y\right)=\frac{1}{\sigma \sqrt{2\pi }}e^{\frac{-{\left(d-d_{\mathrm{rssi}}\right)}^{2}}{2\sigma^{2}}}\;$$

(3) $$d=\sqrt{{\left(x-x_{\mathrm{ap}}\right)}^{2}+{\left(y-y_{\mathrm{ap}}\right)}^{2}}\;$$

where *f*_p_ is the bowl density function, *σ* is a distance variance parameter derived from the 2 dBm variance in RSS, d_rssi_ is the distance obtained from inverting Friis, x and y are the coordinates of a point in the density map, and x_ap_ and y_ap_ are the coordinates of the AP. The form of the bowl function is illustrated in 2D projection and in 3D in Figure 2. The integral of a bowl from a single PR over the x-y plane is, of course, normalized to a total probability of 1.

To illustrate the use of the Bowl Tool, one returns to the toy example, overlaying, in Figure 1b, the bowl densities from each of the three APs that received the emitted PR. In the figure, the resulting sum of probabilities is coded according to a color bar. We note that a red zone corresponding to the region where the “lips” of the three bowls overlap has appeared. In practice, we are free to choose the threshold considered to best circumscribe this region. The decision is now made, analogously to Figure 1a, that the PR has been localized within the red region.

### 4.3. Renormalization Tool

To reflect this decision, the Renormalization Tool is applied by integrating all probabilities outside the decision region and re-injecting them inside the region. The renormalized selected zone appears in 2D in Figure 1c. This defines a small region that is believed to contain a WiFi client. The integrated probability within the region is 3. Since it is clear, in this case, that the three PRs were received simultaneously from a single emission, one divides by 3 to obtain the number of clients, C = 1. Also appearing in Figure 1c is the black star indicating the position determined from the intersection of the three circles in Figure 1a. The two localization methods, i.e., the bowl density accumulation method and the circle-intersection method, are in agreement, as they should be. This agreement between the bowl density measurement and a localization by triangulation is a key element of the approach, as will be detailed in Section 5 and Section 6. The red zone is referred to as a “fiducial region” for two reasons: (a) it is the zone having the highest probability of containing the client; and (b) it is confirmed by triangulation. A return to the notion of a more generalized fiducial region appears in Section 5. In passing, one might have considered multiplying the probabilities of the three APs instead of adding them. In practice, however, this choice proves problematic when dealing with large numbers of APs, some of which may be out of range of one another.

**Figure 3:**
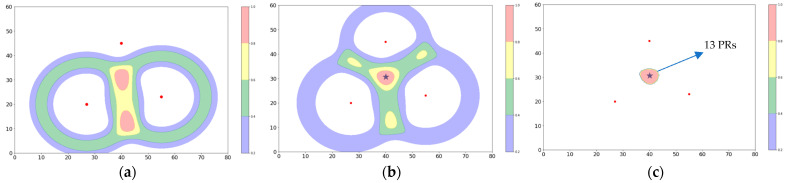
(**a**) Summed probability bowl from 10 PRs emitted by 10 clients, with 5 received at the left AP and 5 at the right AP. The choice of location for the clients is ambiguous (two red zones). (**b**) An 11th client emits a PR that is received by all three APs, breaking the ambiguity. A black star indicates the position of this PR obtained via triangulation. Its position agrees with that of the red zone. (**c**) Renormalizing the probabilities into the central decision region, which has an integrated probability of 13 PRs (6 from the left AP, 6 from the right AP, and 1 from the top AP).

In Figure 3, a new example illustrating some of the difficulties encountered in real-world outdoor WiFi networks is introduced. Consider a cluster of 11 clients with positions randomly distributed over some small region within the triangle formed by the APs. An experiment is performed over a time window in which an average client device would emit a single PR. Implicitly, this refers here to a mean PR emission probability, say X, averaged over all types of devices and client activities, that is, X = 1 for the toy model for the chosen time window. The use of such an X is a core concept of the technique, to which we will refer frequently. For this example, suppose that, due to propagation effects, the average probability of a given AP receiving a client-emitted PR at this distance is about 1 in 3, such that single AP receptions will dominate the experiment, but occasionally two or three APs could be involved. Furthermore, let us suppose that as this time window evolves, five clients each emit a PR that is received only at the leftmost AP, and five others emit a PR that is received only at the rightmost AP. By superimposing the bowls corresponding to these 10 PRs, one obtains the distribution in Figure 3a.

The probability distribution in Figure 3a, at this stage, contains two red zones. Although the clients have slightly different positions, and the noise in their RSS values generates somewhat different bowl radii, the amplitudes of these two peaks are nearly identical, making it impossible to choose the one corresponding to the client cluster. Suppose, however, that before the experimental window closes, the 11th client emits a PR, which, by chance, is picked up simultaneously at all three APs. Adding in the three bowls from these new receptions produces the distribution shown in Figure 3b. It is clear from the figure that the three AP PRs, although a minority occurrence, are sufficient to remove the ambiguity about which peak to choose for the client cluster. Also shown in Figure 3b is the black star obtained by triangulating from the three simultaneously received PRs. Its position confirms the decision made using the density method, as expected. As a caveat, there are clearly a multitude of different configurations of the 11 clients that could have produced the distributions shown in Figure 3. The assumption is that in the limit of larger numbers of clients, as discussed in Section 5, and given the natural tendency of clients to assemble in groups rather than being organized in circles, in symmetric patches, or uniformly distributed over the entire surface of a site, the interpretation chosen is the most plausible one.

### 4.4. Counting Tool

Although the group of 11 clients has been localized in the example, it has not yet been attempted to count them. To do so, the Renormalization Tool may be applied to obtain Figure 3c, where the sum of probabilities of all PR received has been reinjected into the selected red zone. The integrated probability in the zone is then, by construction, 13. If the mean emission probability for the time window, X = 1, mentioned above, is used, the estimated number of clients is C_est_ = 13/X = 13. As the true number of clients is C = 11, it is tempting to say that the estimate is already pretty good. In fact, there are two choices. As a first possibility, if it is certain that the probability of three AP PRs is small compared to that of single AP PRs, one may choose to simply tolerate the counting error they introduce in exchange for the ambiguity-resolution property that the three AP PRs provide. A second possibility would be to measure the fraction of three AP PRs in the data and somehow use it to make a correction to X for a more precise estimate. The Counting Tool and the possibility of introducing such a correction to X will be returned to in greater detail in Section 5 and Section 6.

### 4.5. Filtering Tool

**Figure 4:**
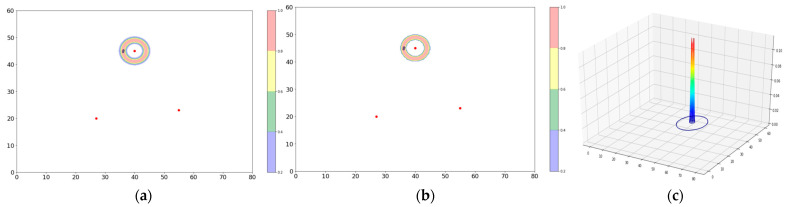
An AP with “indoor-like” propagation characteristics. In (**a**), five clients are in close proximity to the AP, creating a sharply peaked bowl. (**b**) A threshold can be applied to renormalize the density to a more compact region. (**c**) 3D representation of the sharply peaked bowl. The integrated PR count is recorded, and the PRs are removed from the plot to render the smaller-scale structure (shown schematically as a blue circle in the 3D plot) more visible.

Before advancing to the applications in the next section, it is important to discuss a simple, yet troublesome additional configuration frequently encountered in real outdoor WiFi datasets. In some networks, certain APs may be located inside buildings or in other areas favoring very close proximity to the AP—a few meters, for instance. Because of the enclosed geometry and/or accompanying increased distance from the other APs, triangulation is usually not possible. In these cases, the bowl associated with the AP will be very sharply peaked, because of its small radius. As long as the radius is not too large, the consequences for localization are in fact minimal, since one may simply tabulate the total number of PR received—almost exclusively single AP—and associate them with the narrow bowl surrounding the AP location (see Figure 4). The difficulty with using the Bowl Tool here, however, is that the extreme peak often obscures the finer structure of the client density map further away from the AP. For this reason, it is convenient, when analyzing a site with the Bowl Tool, to exclude indoor APs, as well as any PR having RSS > −60 dBm, from the map before continuing the study. This procedure is called the Filtering Tool.

### 4.6. Localization Tool

In the discussion of triangulation, it was assumed that the values of the propagation parameters A and *n* were known. Although, as mentioned earlier, A is normalized in the industry to −30 dBm, it is more prudent to actually measure A for a particular site. Furthermore, the value of *n* is highly dependent on the propagation environment of the site under study. In practice, using incorrect values for A or *n* can lead to dramatically different distance estimations. As site propagation studies are complicated and expensive to carry out, it is often the case in real-world scenarios that reliable ground truth measurements are not available.

**Figure 5:**
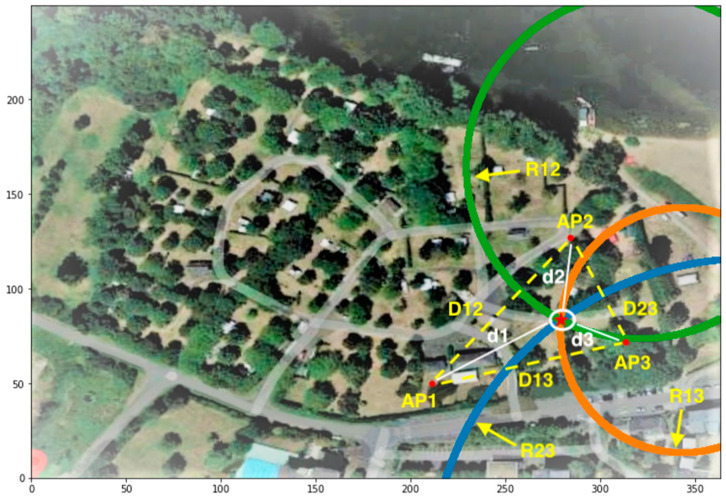
Circles of Apollonius (green, blue, and orange) for AP1, AP2, and AP3, separated by distances D12, D13, and D23. A client within the white circle sits at distances d1, d2, d3 from the three APs. The radii of the circles are R12, R13, and R23. The solution for the client location determined from the RSS values at the three APs is indicated by the red star at the intersection of the circles.

One way to reduce the dependence on propagation parameters is to make use of a simple observation arising from the Friis equation. Consider three APs, AP1, AP2, and AP3, arranged in a triangle as shown in Figure 5, for an example from Site 2. The distances between the APs are D_12_, D_13_, and D_23_. A client located at some position in the vicinity of this triangle—in this case, within it—emits a PR that is received simultaneously at the three APs, with power values RSS_1_, RSS_2_, and RSS_3_. Inverting Equation (1) and taking d_1_, d_2_, and d_3_ as the distances of the client from the three APs gives:

(4) $$\alpha_{\mathrm{ij}}=\frac{d_{i}}{d_{j}}=10^{\frac{{\mathrm{RSS}}_{i}-{\mathrm{RSS}}_{j}}{10n}}\;i\neq j\;=1,\;2,\;3.$$

The dependence of the distance ratios on parameter A has been cancelled out. Equation (2) tells us that the ratio of the distances from the client to any two APs is a constant. As it turns out, this implies that for each pair ij, the client position lies on a circle, called the Circle of Apollonius after the second-century Greek geometer who discovered the relation, which has its center on a line drawn through the two APs and a radius given by:

(5) $$R_{\mathrm{ij}}=\frac{\alpha_{\mathrm{ij}}}{\alpha_{\mathrm{ij}}{}^{2}-1}D_{\mathrm{ij}}\;i\neq j\;=1,\;2,\;3.$$

The circles appear in green, blue, and orange in Figure 5. We stress that these are *not* the same as the triangulation circles introduced in Section 4.1.

If the Friis relation were an exact expression rather than a model, and in the absence of noise on the RSS values and for AP triangles that are not excessively obtuse, any two Apollonius circles will intersect at two candidate client locations, one of which can usually be rejected as unlikely. In more realistic cases, an exact solution may not exist, but numerical solvers can be used to find the best non-exact solution, for example, in a least-squares sense, which is often rather good. The added redundancy of using all three circles instead of just two can also be helpful in such cases. In practice, nonetheless, depending on the site under study, the rate of localizations with poor or no Apollonius solutions can be several tens of percent.

**Figure 6:**
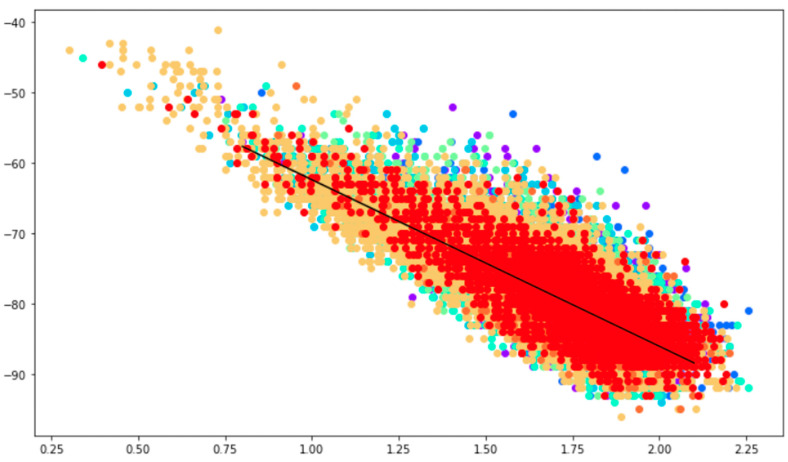
Plot of RSS in dBm versus the log of the distance in meters. Measurement of propagation parameters A and *n* for the Site 1 dataset using Apollonius solutions as client positions. Different colored dots correspond to different APs at the site. A least squares fit to the Friis formula over all APs gives A = 36.2 dBm and *n* = 2.42 for Site 1. Similar results are obtained for Site 2.

The importance of the Apollonius localization solution, apart from being independent of the parameter A, is that the position found also depends only weakly upon the fading exponent *n* for the range 2 < *n* < 3. A simple calculation shows that for *n* in this range, the radius of an Apollonius circle varies by only about 16% of the distance D_ij_ between two APs, which in turn is of the same order as the intrinsic resolution obtainable in outdoor WiFi networks. This relative independence of Apollonius client positions on propagation parameters allows us to envisage using them to calibrate A and *n*, which is a very interesting proposition in the not uncommon real-world situations where propagation ground truth is not available. Figure 6 shows a plot of RSS versus log-distance for the Site 1 dataset, using as distances Apollonius solutions deemed to be of good quality. The resulting distribution is well described by the Friis formula, with A = 36.2 dBm and *n* = 2.24. These values are then used to calibrate the Friis formula for the site in question.

## 5. Application to Real Datasets

In this section, the tools introduced are applied to examples drawn from real WiFi datasets. Additionally, presented are the Fiducial and Drill-Down tools that enable a more detailed and quantitative interpretation of the data reduction process than presented thus far. For each site, a density plot of PR counts over a three-hour period is selected, sufficient to assure a large enough number of accumulated PRs while still allowing time-dependent analysis of client activity. The examples chosen are typical, yet they also illustrate certain particular configurations.

### 5.1. Complete Data Reduction Scenario Applied to Site 1

#### 5.1.1. Applying the Filtering Tool

**Figure 7:**
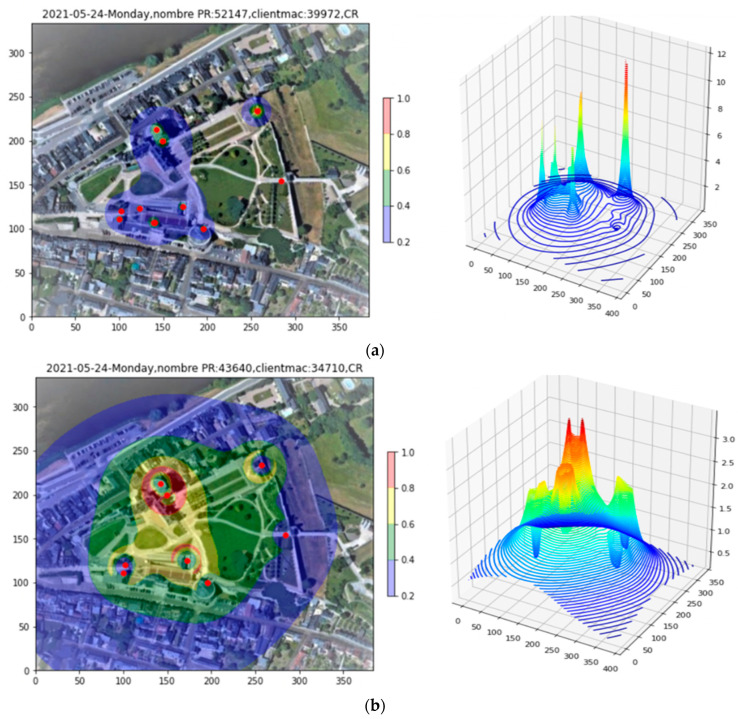
Site 1 PR density plot. (**a**) Raw data. (**b**) After filtering indoor APs and high RSS PRs.

The raw PR density over the three-hour afternoon period from 12:00 to 15:00 is shown in Figure 7, both in 2D and 3D. From the 3D plot, it is clear that sharp peaks caused by clients within a few meters of an AP obscure the finer-scale data. So pronounced is the effect that the 2D plot appears almost empty. To alleviate this problem, the integrated PR count from indoor APs, as well as all PRs with RSS > −60 dBm, is recorded, and then these PRs are removed from the plot (Filtering step). The density after filtering appears in Figure 7b), where the underlying structure of the density over the full site begins to become apparent.

#### 5.1.2. The Fiducial Tool

The red regions in Figure 7b correspond to higher “bowl”-based probabilities of the presence of clients. In principle, one could apply a threshold and renormalize the probabilities, as in Section 4.3, but the problem is more complex now. There are 8 APs and more than 43,000 PRs represented in the plot. In addition, there are still some “peaky” structures apparent. What is the best threshold to apply here? In order to reply with more confidence, the bowl density information is complemented with independent confirmation from localizations based on PRs received simultaneously at three APs. Although these represent only a fraction of the total PRs and the efficiency for successful Apollonius localization, as discussed in Section 4.6, falls well short of 100%, if there are enough cases of three AP PRs, their spatial localizations will be useful for confirming the information provided by the density itself.

**Figure 8:**
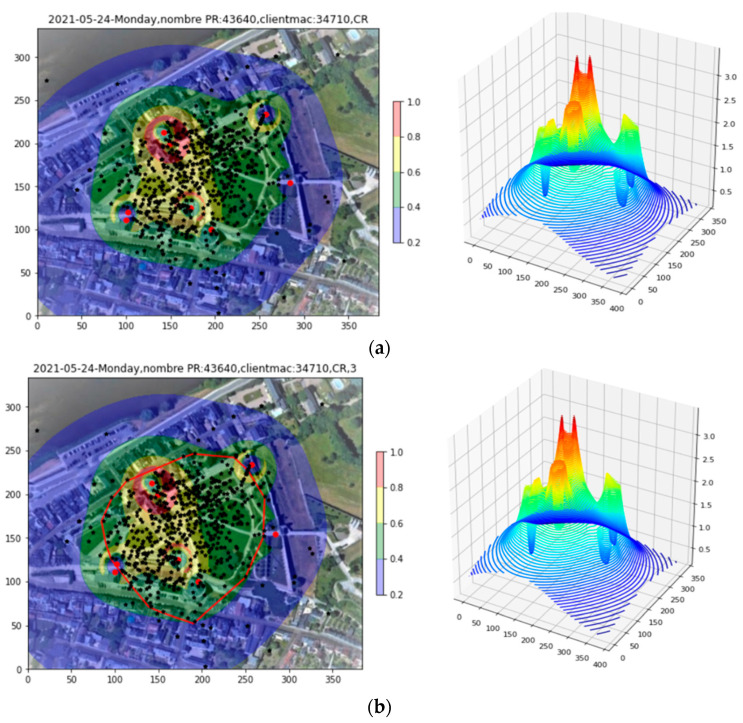
(**a**) The successful Apollonius localizations are added to the density plot as black stars. Convex layers are next tested as candidate fiducial regions. (**b**) The fifth convex shell is most appropriate for eliminating outliers while retaining the major concentration of points.

Figure 8a shows the density plot of Figure 7b with the successful Apollonius localizations superimposed upon it as black stars. There are about 700 of them, some of which appear outside of the covered site. It is desired to characterize the region occupied by these localizations so that it may be used as input to the Renormalization Tool. In order to select a region that represents the bulk of the localization while eliminating outliers, use is made of the Convex Layer Set of the localization point distribution. The first layer of this set is the Convex Hull; the second is the convex hull of points remaining after removing the first hull; and so on. Since there are many localizations here and a significant number of outliers due to poor-quality Apollonius solutions, the fifth Convex Layer is chosen to bound the points, as shown in Figure 8b. This use of Apollonius localizations and Convex Layers to characterize the true spatial extent of the client density is called the Fiducial Tool.

#### 5.1.3. Applying the Renormalization Tool

**Figure 9:**
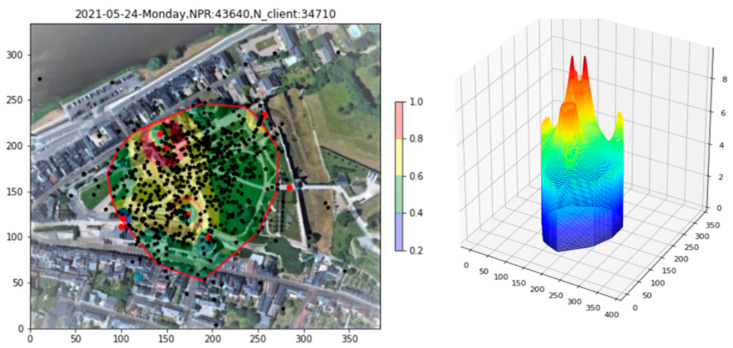
Renormalization of probabilities to the interior of the fiducial region.

With the fiducial region now defined, all probabilities are renormalized into the region. The result is shown in Figure 9, both in 2D and 3D. All densities outside the fiducial region have disappeared, allowing us to focus on the region in which the clients are actually located. Within the fiducial region, in the 2D plot, one may note two regions of enhanced probability, seen as peaks in the 3D plot. At the same time, these “bowl”-based enhancements are not confirmed by an enhanced density of Apollonius points beneath them. Indeed, the cylindrical nature of the 3D peaks points to PR reception predominantly by single APs.

#### 5.1.4. The Drill-Down Tool

**Figure 10:**
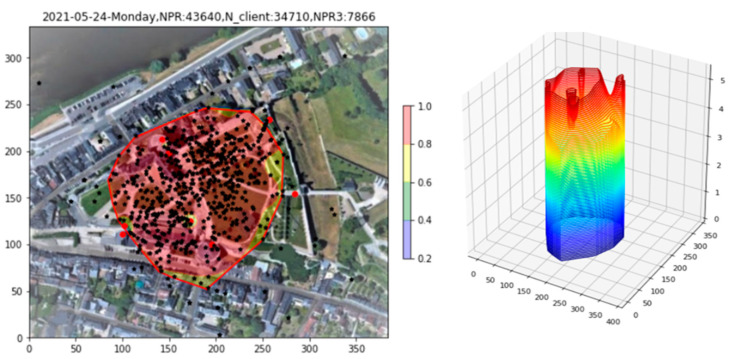
Drilling down on Site 1 to reveal the density structure on a finer scale. The uniform density distribution is supported by the uniform distribution of localizations (black stars). The threshold applied still retains 90% of the total PRs. PRs removed by the thresholding will be added back in later.

In order to minimize the effect of these peaks and better study the behavior of the density plot at smaller scales, a threshold is progressively lowered—easily controlled, for example, with the wheel of a mouse—that drills down to a level where the finer structure of the density becomes apparent. The PRs thus “decapitated” from the peaks are stored for later reincorporation (as discussed in the next section). The result, displayed in Figure 10, shows a bowl density that is nearly uniform—and therefore now in conformity with the uniform distribution of localization points. Note that the chosen threshold still retains 90% of the total PR. The analysis of the site 1 example is now complete. What remains, reassembling the different pieces into a coherent map, appears in Section 6, after the presentation of the example from Site 2.

### 5.2. Complete Data Reduction Scenario Applied to Site 2

A second example, from Site 2, is presented here, illustrating a different situation.

#### 5.2.1. Applying the Filtering Tool

**Figure 11:**
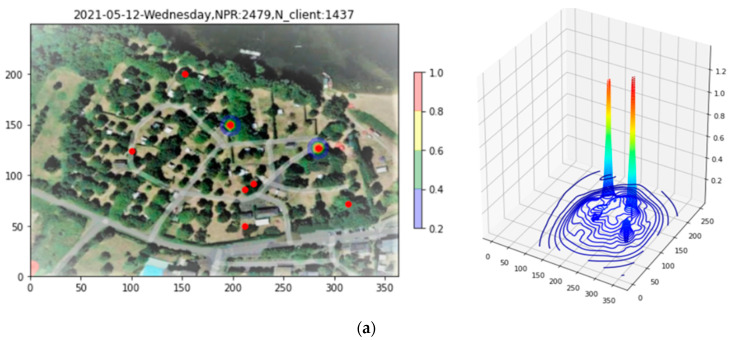

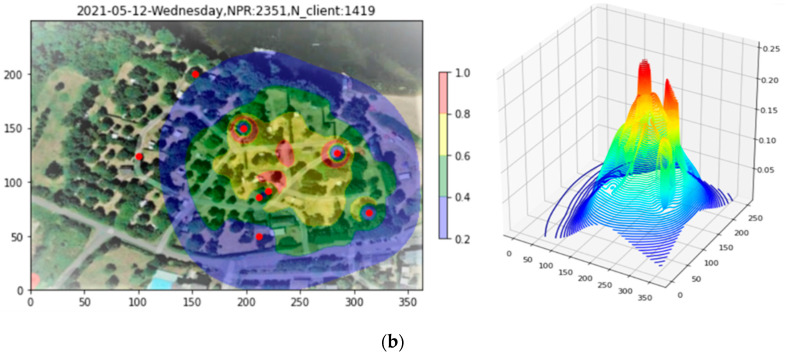
Application of the Filtering tool to the density plot of Site 2. In (**a**), two peaks due to high-RSS PRs dominate the plot. Removing these, (**b**) reveals the finer structure of the density.

In Figure 11a, it is to be noted that the three-hour period studied here, this time for the evening period of 18:00 to 21:00, presents only 2479 PR, almost a factor of twenty less than the example presented for Site 1. A second observation is that, as for Site 1, the plot is dominated by narrow peaks, two in this case, due exclusively to PRs having RSS > −60 dBm. In Figure 11b, these PRs are removed with the filtering tool, revealing the finer structure.

#### 5.2.2. The Fiducial Tool

**Figure 12:**
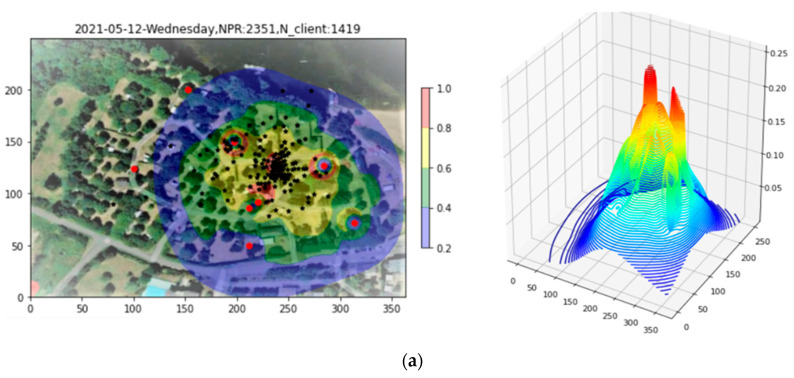

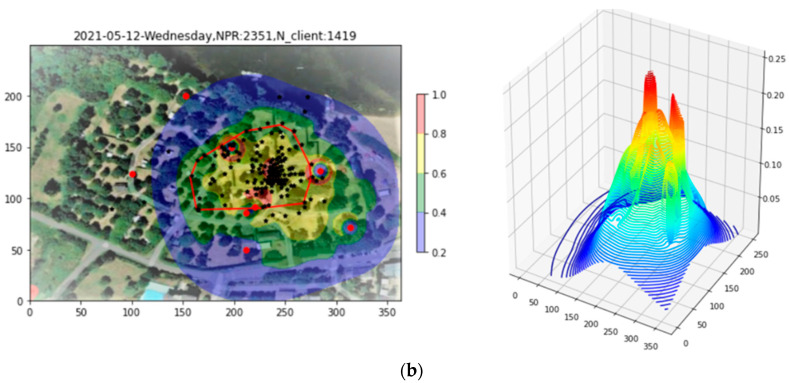
(**a**) Site 2 density plot with Apollonius points superimposed. (**b**) The third Convex Layer applied to delineate the fiducial region.

Once again, it is desired to restrict the study to a fiducial area likely to represent the region where the bulk of clients are actually located. Figure 12a shows the Site 2 density plot with the Apollonius points, of which there are some 200, superimposed. In this example, with a much lower PR count, the third Convex Layer is sufficient for delineating the area of interest and excluding outliers, as shown in Figure 12b.

#### 5.2.3. Applying the Renormalization Tool

**Figure 13:**
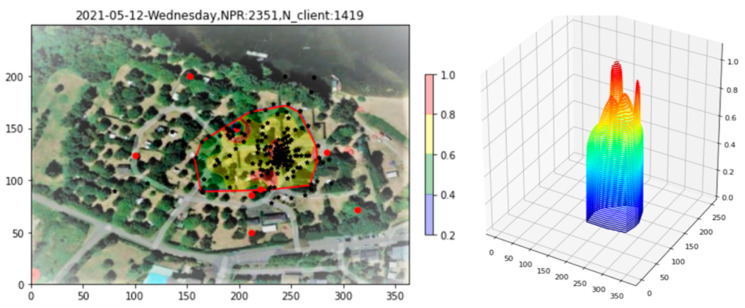
Renormalizing the Site 2 density into the fiducial region. The dominating features are a peak associated with a single AP, upper left of the fiducial region, as well as two additional small regions at the center and lower center that coincide with an enhanced density of Apollonius localizations.

In Figure 13, the Renormalization Tool is applied to the Site 2 example. Three key features in the resulting bowl plots should be noted, perhaps more easily visible as small red zones in the 2D representation. The first is a cylindrical peak associated with a single AP in the upper left central part of the fiducial region. The second and third are two small red zones, near the center and lower center of the fiducial, that coincide with enhanced densities of localization points—in contrast to the Site 1 results, where the Apollonius density was relatively constant over the fiducial region.

#### 5.2.4. The Drill-Down Tool

**Figure 14:**
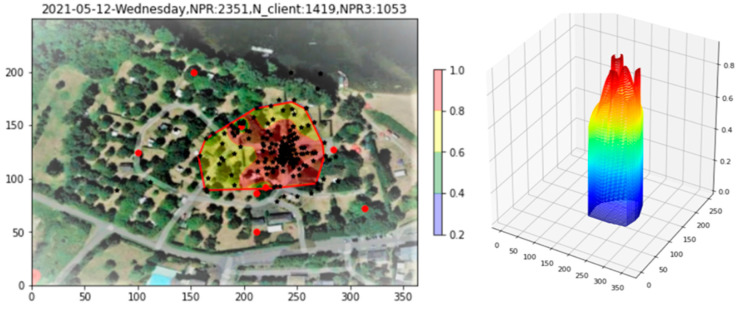
Drill-Down tool applied to renormalized Site 2 density. One observes a red zone coincident with high localization density, and a yellow zone coincident with lower localization density.

To further study the relationship between the bowl-based density and the density of Apollonius localizations for this site, let us now apply the Drill-Down Tool. The result is shown in Figure 14. The drill-down performed retains 99.7% of the total PRs; its effect has been to just slightly reduce the tallest peak in the plot. With this threshold, however, in the 2D plot, the higher Apollonius densities are now contained in a red, enhanced bowl density zone, while the lower Apollonius densities are in the yellow part of the fiducial region. This observation alerts us to a possible source of error if one were to use a single proportionality factor X to convert from PR to clients, since the red region contains a higher percentage of clients whose PRs are seen by three APs. To take this effect into account, in Section 6, some improvements to the Counting Tool are introduced before detailing the Reassembly Tool, which outputs final site maps calibrated for clients rather than PR, is presented.

## 6. Reassembly Tool

As stated earlier, the purpose of the Reassembly Tool is to put together the different pieces of the PR density map into a map calibrated for clients rather than PR. The idea that client counts are obtained from PR counts by dividing by a proportionality factor X appropriate to the time window in question has already been introduced. In order to follow client activity over time, however, the focus is placed on blocks of three hours throughout the day, which may need different values of X according to the type of activity—morning, afternoon, weekend, active, resting, etc.—prevalent in each block. Evidence is also visible, in the example of Site 2, of X values that vary according to the position, in particular regarding the percentage of PRs that are simultaneously detected at three APs and can thus be localized. In order to correctly reassemble the different pieces of the PR density map into a coherent client density map, one must therefore possess two key elements: a baseline X value for the site under study and a technique for correcting X as a function of time and position on the density map. In the next section, some extensions to the Counting Tool designed to facilitate the reassembly process are proposed.

### 6.1. Counting Tool Revisited

#### 6.1.1. Determining a Baseline Site X Value

As mentioned earlier, the data used in this work are part of a larger set that includes the city data reported in [1]. The principle contribution of that work was a means of deriving baseline X values for a site from statistical properties of its PR data, in particular, weekly periodicities in workday versus weekend populations present in the city data. In [1], for three French cities over two distinct time periods in 2020 and 2021, measured *daily* X values ranged from 420 to 670 (where a typical error bar is 10–15%), with a mean over all sites of X = 524 ± 47 [1], again, for a period of 1 *day*. However, as the data in the present work were acquired at a historic tourist site (Site 1) and a campground (Site 2), their PR counts *do not* manifest the clear weekly periodicities that were the norm for the city data and, as such, cannot be directly used to measure X for the two new sites using the statistical method developed in [1].

It is, however, possible to demonstrate that the X values for the two current sites, after certain adjustments, are consistent with those measured in the city data. Indeed, in [1], the measured city X values were validated against several independent benchmarks, including comparisons to similar measurements in the literature, as well as a calculation based on the connected clients (CC) present in those data. The idea is that since the MAC addresses of CC are fixed, one may directly count the number of PRs per CC. As a caveat, one does not a priori expect the behavior of CC to be identical to that of CR. In particular, CC often make use of the fixed WiFi connection to set up Extended Services or ES (see [1]), such as Peer-to-Peer networks, which can produce extremely large PR counts. However, by eliminating clients considered to be participating in ES [1], one expects the X values obtained for CC and CR to be relatively similar, as was the case in [1].

For the city data in [1], the CC-estimated X values were obtained using the relation [1] X_CC_ = P/(A_CC_<t_CC_>), where X_CC_ is the estimated X value for the CC, P is the number of PR, A_CC_ is the number of CC, and <t_CC_> is their average duration of stay on the site during the chosen time window. For the data in [1], the resulting X_CC_ values ranging from 378 to 948 are in reasonable agreement with the CR-estimated X range in [1] mentioned in the preceding paragraph, namely 420 to 670. One may say that in [1], the CC were used to validate the X values obtained from the CR in [1]. To now validate the CR data of Sites 1 and 2 in the present article, using the CC of the present article, one proceeds in the identical way. The results are given in columns 1 and 2 of Table 1.

The first column of the table gives the percentage of CC retained after the ES-cut. For Site 1, very few clients had abnormally large PR emission rates, while for Site 2, the percentage was much higher. It is supposed that this is due to the different characters of the two sites. At a historical tourist site such as Site 1, most guests will stay only a short time and spend most of it shuttling from one point of interest to the next, and as such, they will have little incentive to connect to the local WiFi to set up special situations. Site 2, however, is a campground that includes bungalows. Guest will often stay several days in quarters that may lack the wireless environment and tools to which they were accustomed at their usual domiciles, so the motivation for piggybacking off the local WiFi—with its potential for high PR count services—will be high. Furthermore, at both sites, OUI values of the high-PR count CC, when exploitable, often pointed to IoT-type devices rather than user telephones. Finally, we stress that the overall number of CC is a small fraction of that of CR, making it less exploitable statistically for a detailed study, a point that will be important in the discussions that follow.

The second column of Table 1 gives the mean baseline X values of the CC in the present work, calculated using the formula presented above, X_CC_ = P/(A_CC_<t_CC_>), along with their Mean Absolute Deviation, MAD. It is seen that the mean value of 369 for Site 1 fits well with the range of CR-based X values (420–670) as well as the X_CC_ values (378–948) obtained for the cities in [1]. As for Site 2, the situation is somewhat different. The X value obtained from the CC here, 1139, is rather high compared to the others. In addition, its MAD of 394 seems curiously large compared to the normally encountered range of 10–15%, mentioned earlier. The next section will show that these differences are a result of the behavior of P_3_, the number of PR received at three APs, in the present data as compared to the city data in [1].

#### 6.1.2. A Strawman: Site-Dependent, Time-Dependent X_base_

As mentioned in Section 3, the class CR in this article was defined by requiring no more than three observations of a hashed MAC address in the selected time window. This threshold ensures that most of the CR MAC addresses are indeed randomized—i.e., do not retain the same MAC address over several PR emissions—but still retains the case when a CR client’s PR is observed at three APs, enabling this client’s PR to be localized via triangulation. In contrast, for the city data analyzed in [1], a threshold of 2 was selected. Although this choice also ensures a clean separation between CR and CC, it removes the possibility of triangulation. Indeed, it was found empirically that three AP hits were very rare in the city data of [1]: only about 2% of PRs were detected twice in the course of a day, and a tiny additional proportion at three APs. This is in stark contrast to what is observed for Sites 1 and 2 of the present work, where three AP hits can make up a substantial fraction of total APs. Indeed, were there not at least some fractions of CR seen simultaneously at three APs, it would be considerably more difficult to speak of localization at the two Sites. This underlying difference between the city data and that of Sites 1 and 2 can be attributed to the geometrical layout of the APs in the two cases. In the city data of [1], the deployed networks invariably consisted of a limited number of widely spaced APs arranged in predominantly linear configurations ill-suited for triangulation.

In order to put the city data and Sites 1 and 2 data on an equal footing for comparison, as well as provide a means to take into account the increased P_3_ values at Sites 1 and 2, we propose the following “strawman” procedure. As a first-order approximation, one may assume that the X reported in [1], which we shall now call X_12_, is valid for sites where multiple AP PRs are rare and, from there, attempt to make a correction for sites having more dense AP layouts and hence, more three-AP PRs. We pose the following:

(6) $$P\;\sim {\;P}_{12}+P_{3}\;\sim {\;C}_{12}X_{12}+3C_{3}X_{12}=\left(C+2C_{3}\right)X_{12*}\;$$

(7) $$X_{\mathrm{base}}=\frac{P}{C}=(1+\frac{2C_{3}}{C})X_{12}=(1+\frac{2\frac{P_{3}}{3X_{12}}}{C})X_{12}=(1+\frac{2\frac{P_{3}}{3X_{12}}}{\frac{P}{X_{\mathrm{base}}}})X_{12}\;$$

(8) $$X_{\mathrm{base}}=X_{12}+\frac{2}{3}\frac{P_{3}}{P}X_{\mathrm{base}}\;$$

(9) $$X_{\mathrm{base}}=\frac{X_{12}}{1-\frac{2}{3}\frac{P_{3}}{P}}\;$$

where P is the total number of PRs, C is the total number of clients, P_3_ is the number of PRs seen simultaneously by three APs, C_12_ is the number of clients producing one or two PRs per emission, and C_3_ is the number of clients whose PRs were seen at three APs. Note that in the last line of Equation (9), X_base_, the corrected site baseline X value, is expressed completely in terms of measurable quantities P and P_3_, and X_12_ from [1], assumed to be valid also for the sites studied here. For P_3_ = 0, we retrieve X_base_ = X_12_, as required, while for P_3_ = P, the strawman predicts X_base_ = 3X_12_, which is reasonable given the initial assumptions.

One may get an idea of the applicability of the strawman by referring again to Table 1. In column 3 are presented the range of values of the ratio P_3_/P for Sites 1 and 2. It is evident that the absolute numbers of CC at Sites 1 and 2 are too low to provide a statistically stable measurement of P_3_/P. To remedy this, in columns 3, 4, and 5 of the table, the ranges and mean values of P_3_/P measured for the CR are cited, which should be similar to those of the CC since P_3_/P is a quantity that depends predominantly on network layout. Columns 3 and 4 of the table show that the fraction P_3_/P is indeed significant and that it is much larger at Site 2 both in magnitude and in range. This observation is consistent with the much increased MAD value at site 2, i.e., the fraction P_3_/P varies significantly throughout the course of a day, leading to large variations about the mean value. In column 5 of the table, this average P_3_/P value is used to calculate the reciprocal of the strawman fraction, thus creating an ad hoc correction factor that allows to put the Site 1 and 2 daily X values on a common footing with those of the city data in [1], for comparison. In column 6, the resulting values are indeed now seen to be coherent with the ranges mentioned earlier, i.e., 420–670 for the measured city values and 378–948 for the CC-based validation values.

As a caveat, it must be remembered that the strawman prescribes using the *instantaneous* value of P_3_/P, not its mean, to correct X_12_. Column 6 of the table is intended to show the general trend of the correction. When actually used in the counting and reconstruction tools, the instantaneous values of CR-derived P_3_/P values are to be used. It is also for this reason that in the following, we prefer to use X_12_ = 524 from [1] as the canonical conversion factor, rather than the CC-derived, ad hoc corrected values reported in column 6 of Table 1.

It is clear that P and P_3_ are site-dependent quantities, so X_base_ automatically adapts to each site. Also, in the proposed treatment, the time window used is not explicitly specified. Indeed, the resulting formula for X_base_ is valid for P and P_3_ from any time window. In particular, it is applied directly to the 3-hour windows chosen for this article, so that X_base_ also adapts to each individual time window. As a caveat, the constant value X_12_ in the above equations must be adjusted to correspond to the three-hour time window chosen for the client density plots in the present work, i.e., X_12_ = 524 × (8/24) = 65.

In creating the strawman, some simplifying approximations have been made. Clearly, a more rigorous treatment is possible—for example, by explicitly including a term for P_2_ or P_4_, etc.—but given the statistical uncertainty in X_12_ to begin with and the systematic uncertainties in applying it to new data sets, this may not be the major priority at this stage. The strawman provides an informed, first order estimation of the results that may be expected in estimating numerical client densities from raw probe requests in outdoor WiFi networks for which ground truth is not available. We shall employ it in the Reassembly Tool, where appropriate, for estimating overall client counts versus position.

#### 6.1.3. X_base_ Adapted for Position

In Section 5.1.4, we identified red and yellow zones in the bowl density that coincide with different densities of Apollonius localizations, that is, an enhancement in the number of localizations in the red zone and a relative deficit in the yellow zone. In the same discussion, it was suggested that the bowl density in the red zone—which counts PRs—may have been artificially enhanced due to the abundance of PRs seen at three PRs. Because of the way the bowl density is constructed, it is not possible to know the numbers of single-AP and three AP PRs in the red and yellow zones. However, as a substitute, it is straightforward to count the number of localization points in the two zones. It is proposed to use this information to correct X_base_, color zone by color zone, when reassembling the different pieces of each site, as outlined in Section 6.2 below for the two sites studied. The position-dependent correction to X_base_ may be applied according to:

(10) $$X_{\mathrm{color}}=\frac{X_{12}}{1-\frac{2}{3}\frac{{P_{3}}^{\prime }}{P}}{{;\;P}_{3}}^{\prime }=\frac{N_{\mathrm{Apollo}}\left(\mathrm{color}\;\mathrm{zone}\right)}{N_{\mathrm{Apollo}}}P_{3}\;$$

where N_Apollo_(color zone) is the number of Apollonius localizations in each color zone of the bowl density plot, and N_Apollo_ is the total number of such localizations.

A difficulty with this correction as it stands is that while it adapts X to the different color regions, it will alter the overall predicted number of clients. This problem can be circumvented by normalizing the X_color_ values with

(11) $$\langle \frac{1}{X_{\mathrm{color}}}\rangle =\frac{1}{P}\underset{i}{\sum }(P_{i}\cdot \frac{1}{X_{i}})\;$$

(12) $$X_{\mathrm{color}}\leftarrow X_{\mathrm{color}}\cdot X_{\mathrm{base}}\cdot \langle \frac{1}{X_{\mathrm{color}}}\rangle \;$$

An example, with the values obtained for Site 1 in Figure 14 is given in Table 2.

The seventh and last column of the table gives the values of X_color_ obtained from Figure 14, while columns 4, 5, and 6, respectively, give the estimated number of clients using the fixed legacy X value from [1] normalized to a 3-hour period (65); a fixed overall site P_3_ corrected X value (93); and a color zone by color zone correction (values appearing in the last column). The estimated numbers of clients vary depending on the X method used, as expected. As an example, the prediction for the yellow region is 13.9 clients for uncorrected legacy X (column 4), 9.7 clients for an overall site-corrected X value (column 5), and 11.2 clients when X_color_ is used to correct for position (column 6). What has been done, using X_base_ and X_color_, is to correct for the artificial enhancement in bowl density, using legacy X, caused by the presence of proportionally more three-AP PRs.

### 6.2. Reassembly Tool

#### 6.2.1. Reassembly of Site 1

**Figure 15:**
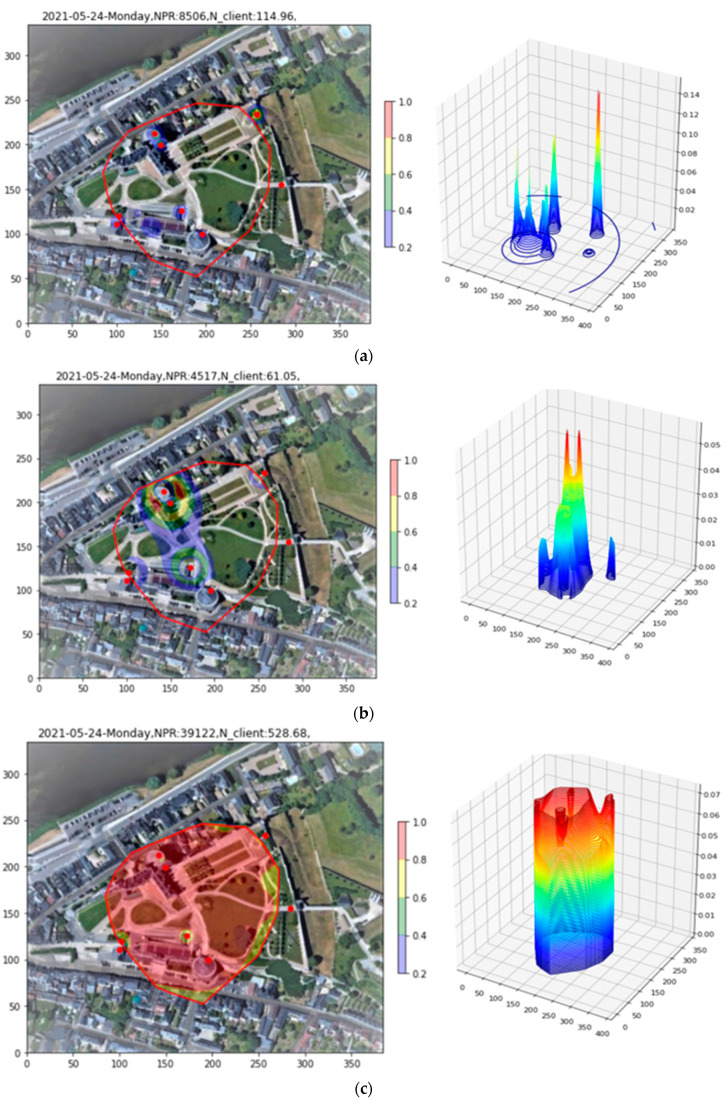
Site 1. (**a**) Filtered indoor APs and high-RSS PRs; (**b**) peaked structures removed by Drill Down; (**c**) Density retained after Drill Down.

The Site 1 PR density removed by filtering and drilling down, as well as the PR density retained after drilling down, are shown graphically in Figure 15, recapitulating some of the steps already seen in Figure 10. As the post-drill-down PR density shows no particular structure, it is not necessary to apply position-dependent X. The client density is then obtained by dividing all the pieces by the global site X factor of the time window and summing the results.

**Figure 16:**
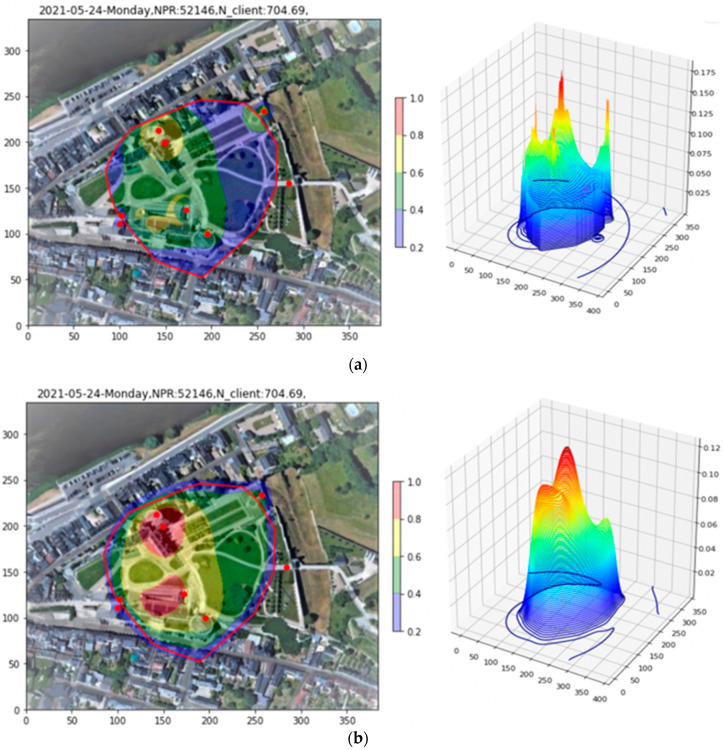
Site 1. (**a**) X-weighted sum of pieces. In this case, all pieces are weighted by the global site X value for the time window. (**b**) X-weighted sum after smoothing by a Gaussian kernel having a standard deviation of 10 m to smooth sub-resolution artifacts (peaks, edges, etc.).

The resulting client density map appears in Figure 16a. To make the plot more readable, the density is smoothed with a Gaussian kernel of standard deviation 10 m, of the same order as the intrinsic position resolution, in order to remove sub-resolution scale features such as narrow peaks and boundaries between regions (see Figure 16b).

In the 2D plot, two red high-occupancy zones are visible. The estimate for the lower one, which is consistent with the site entry ticketing area, is 68 clients on a surface area of 1990 m^2^. The upper one, corresponding to one of the major tourist attractions of the site, is estimated to contain 48 clients in an area of 1482 m^2^, while the surrounding yellow zone contains 245 clients in an area of 8954 m^2^. The total integrated number of clients in all regions of the plot is estimated at 704.

#### 6.2.2. Reassembly of Site 2

**Figure 17:**
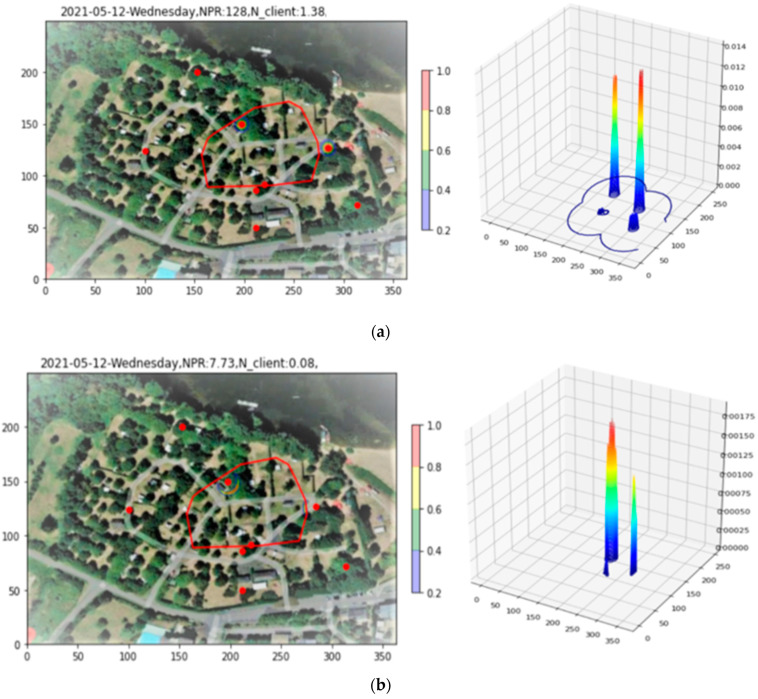

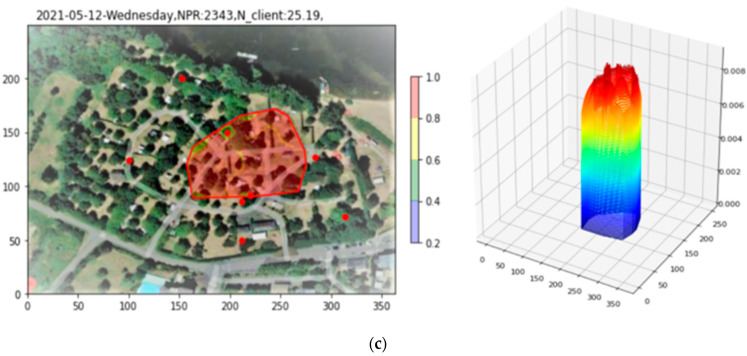
Site 2. (**a**) High-RSS PRs removed by filtering; (**b**) peaked structures removed in drill down; (**c**) densities remaining after drill-down weighted by position-dependent X and summed.

**Figure 18:**
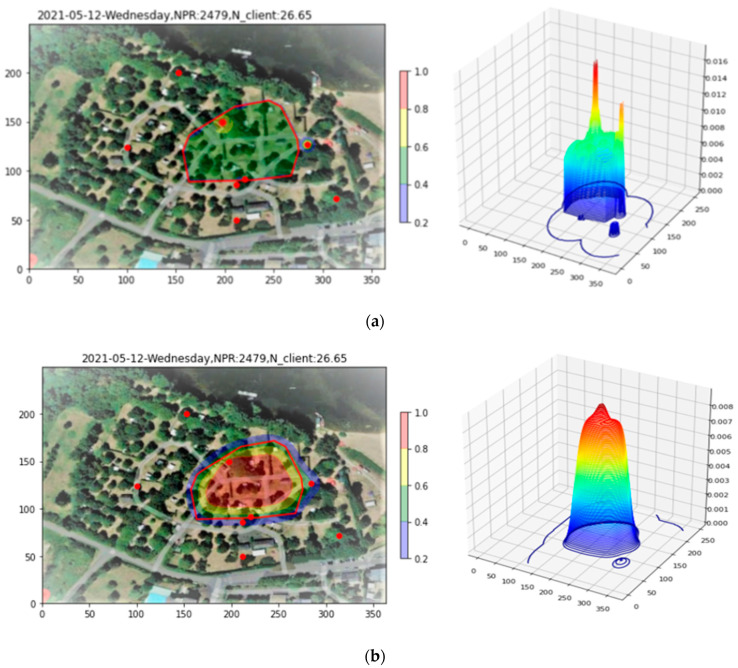
Site 2. (**a**) X-weighted sum of pieces. Pieces are weighted by site X for the time window and corrected by position on the map. (**b**) X-weighted sum after smoothing with a Gaussian kernel standard deviation of 10 m.

The same procedure is followed for Site 2, as in Figure 17a,b, except that the after-drill-down density, Figure 17c has been corrected by a position-dependent X, as explained in Section 6.1. The after-drill-down density in Figure 17c is more uniform now compared to that in Figure 14, due to this density-dependent X correction. The total summed density is shown in Figure 18, before and after smoothing with a Gaussian kernel of standard deviation 10 m in order to remove sub-resolution scale features such as narrow peaks and boundary lines.

**Figure 19:**
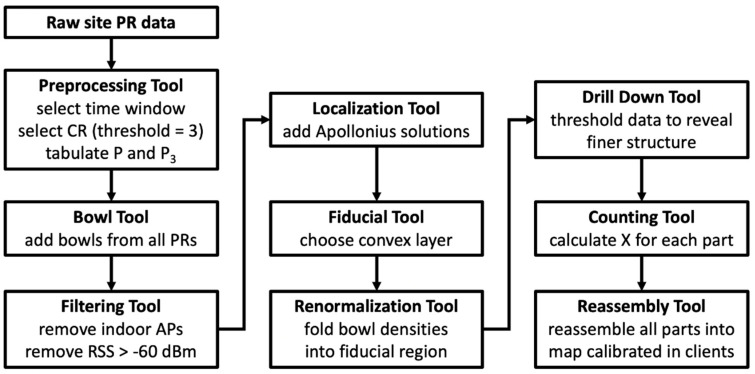
Flow chart resuming the entire data reduction protocol using the toolkit.

Our technique estimates that in the red zone of Figure 18a, there are 16 clients in a surface area of 3741 m^2^, while in the surrounding zone, there are 7 clients over 2124 m^2^. The client estimate for the entire plot is 33. A flowchart resuming the different steps used in applying the toolkit is given in Figure 19.

As we have seen, the “Bowl,” Filtering, and Drilldown tools are graphical techniques used to make the spatial representation of client density clearer. The Reconstruction tool, in turn, in concert with the others, is a bookkeeping tool that allows one to retrieve the different pieces into which the problem has been divided and reconstruct them into a coherent and easily interpretable whole. It is the Fiducial tool, making best use of the limited localization information available in low-resource networks; the Localization tool, calibrated in a bootstrap procedure; the multi-step Counting tool allowing time and space domain adaptation of the X proportionality factor; and the Renormalization tools for redepositing the PR probabilities within the deduced fiducial region, that embody most of the originality and warrant the success of the approach presented.

## 7. Discussion and Conclusions

With the arrival of client privacy concerns as well as the rapid growth of IoT, the widespread practice of using client MAC addresses contained in PRs for monitoring and mapping client activity has become untenable, creating a need today for new tools. Here, a set of nine tools is presented to transform raw randomized MAC PR counts from real-world outdoor WiFi networks directly into density maps calibrated for clients, all without the use of any ground truth. The technique has been applied to data from two actual network sites in France, with interesting results.

At present, the density predictions of the technique make use of a baseline PR emission probability, X, adopted from a similar study, augmented with a few reasonable, yet approximate, corrections. A high-priority perspective is thus to discover more precise estimation methods and use them to evaluate our technique on current data. An important route towards this goal is to encourage interactions with outdoor WiFi service providers and site managers so as to evaluate and calibrate the tools proposed here.

Indeed, personnel having day-to-day familiarity with the details of monitored sites are likely to possess precious expert information not easily available to the data scientist, armed with an Excel file and a Google Maps satellite view of the site, charged with extracting the necessary metrics. Several examples are given as follows:

- Local network managers will probably retain records of the site IoT devices, such as cameras, lighting, loudspeakers, etc., in regular use, as well as of user equipment assigned to site personnel and potentially participating in P2P or similar services. Such information will be invaluable for separating the corresponding PR counts of these devices from those derived from bona fide clients.
- Site managers will also likely possess information on seating capacities of auditoriums, attractions, etc.; guest capacities of lodging and restaurant facilities; and visitor capacities of attractions, events, and the site itself. Such limits can be used to constrain estimates of the local site X factor.
- In cases where complementary footfall counting equipment is present at certain locations on a site, for example, head counts, presence sensors, etc., their outputs can also be used to condition estimates of the PR to client conversion factor X.
- Personnel familiar with site layout and maintenance will also have knowledge of land occupation distribution. Areas that are inaccessible, cordoned off, or fenced off, restricted in access, or simply situated outside the detailed official boundaries of the site, can be excluded from the fiducial area when the Renormalization tool is applied. Too, special-use areas, such as gardens, paths, parking lots, playing fields, and golf courses, will probably display client behaviors particular to the special usages of these areas and can be treated separately from more general user access areas when predicting client densities.
- When available, gate receipts, ticket counts, etc. may also, in certain circumstances, serve as indirect ground truth to which WiFi-derived client counts can be compared.
- Finally, site managers and organizers will also have access to scheduling information concerning day-to-day, holiday, and seasonal opening hours for the entire site as well as for individual facilities, including swimming pools, restaurants, golf, etc., and for special events as well as temporary, occasional, or unscheduled closures. Such timetables will be extremely valuable in establishing coherence between WiFi-derived counts and ground-truth expectations.

The mapping of client activity based on randomized MACs is still a new endeavor. The simplicity and independence from ground truth of the proposed toolkit make it an interesting new contribution to the ongoing activity in the field, both for researchers and for the personnel involved in managing the sites under study.

## Figures and Tables

**Table 1:** X validation results using CC, for the present article, evaluated for a *1-day period* to allow direct comparison with [1]. Column 1: percentage of CC retained after the ES-cut (see text). Column 2: baseline X value computed using P/(A_CC_<t_CC_>) as discussed in the text. The value for Site 1 compares well to results for cities in [1], but that for Site 2 is too high and has a large variance (see discussion in text). Column 3: approximate range of the values, or P_3_/P in the CR data of the two sites. Column 4: mean value, of P_3_/P in the CR data of the two sites. Column 5: correction to the value in column 2 using the formula derived in the text from P_3_ and P. Column 6: predicted X12 values directly comparable to X values from the city data in [1]. After the correction, the mean values in column 6 compare well with the city data in [1]. The large variance of the Site 2 value is discussed in the text.

Site	% of CC after ES-Cut	X_base_ (for CC)	$\mathrm{Range}\;\frac{P_{3}}{P\;}$ (for CR)	$<\frac{P_{3}}{P\;}>\;$ (for CR)	Correction $1-\frac{2}{3}\langle \frac{P_{3}}{P}\rangle$	Predicted X_12_ (for CC)
1-historic	99.8%	369 ± 36	0.0–0.15	0.07	0.953	352 ± 34
2-camping	92%	1139 ± 394	0.2–0.7	0.35	0.767	873 ± 302

**Table 2:** Breakdown of X_base_ by color zone and corresponding number of clients for Site 2 for the drill-down threshold in Figure 14. Columns are %pr: percentage of total PR; Nb_pr: number of PR; Nb_apollo: number of Apollonius localizations (black stars); the next three columns are Nb_client: estimated number of clients using three estimates of X; and in the last column, the color-by-color X_color_ values from the plot.

	%pr	Nb_pr	Nb_apollo	Nb_client (X_12_ = 65)	Nb_client (X_base_ = 93)	Nb_client (X_color_)	X_color_
total	99.67	2343.27	163	36.05	25.2	25.2	77.49
red	59.21	1391.99	135	21.42	14.97	13.43	86.36
yellow	38.45	903.97	20	13.91	9.72	11.16	67.47
green	2.01	47.3	8	0.73	0.51	0.6	65.97
blue	0	0	0	0	0	0	65
transparent	0	0	0	0	0	0	65

## Data Availability

Restrictions apply to the availability of the data used in this study, which is third party data provided by Aleia. Requests for access to the data should be addressed to the corresponding author and must be approved by Aleia.
